# Detection of SARS-CoV-2 Infection in Human Nasopharyngeal Samples by Combining MALDI-TOF MS and Artificial Intelligence

**DOI:** 10.3389/fmed.2021.661358

**Published:** 2021-04-01

**Authors:** Meritxell Deulofeu, Esteban García-Cuesta, Eladia María Peña-Méndez, José Elías Conde, Orlando Jiménez-Romero, Enrique Verdú, María Teresa Serrando, Victoria Salvadó, Pere Boadas-Vaello

**Affiliations:** ^1^Research Group of Clinical Anatomy, Embryology and Neuroscience (NEOMA), Department of Medical Sciences, University of Girona, Girona, Spain; ^2^ICS-IAS Girona Clinical Laboratory, Santa Caterina Hospital, Parc Sanitari Martí i Julià, Salt, Spain; ^3^Science, Computation, and Technology Department, School of Architecture, Design, and Engineering, European University of Madrid, Madrid, Spain; ^4^Instant Biosensing Technologies, Carson, NV, United States; ^5^Analytical Chemistry Division, Department of Chemistry, Faculty of Science, University of La Laguna, La Laguna, Spain; ^6^Department of Chemistry, Faculty of Science, University of Girona, Girona, Spain

**Keywords:** MALDI-TOF MS analysis, machine learning, SARS-CoV-2, NP samples, viral transport media

## Abstract

The high infectivity of SARS-CoV-2 makes it essential to develop a rapid and accurate diagnostic test so that carriers can be isolated at an early stage. Viral RNA in nasopharyngeal samples by RT-PCR is currently considered the reference method although it is not recognized as a strong gold standard due to certain drawbacks. Here we develop a methodology combining the analysis of from human nasopharyngeal (NP) samples by matrix-assisted laser desorption/ionization time-of-flight mass spectrometry (MALDI-TOF MS) with the use of machine learning (ML). A total of 236 NP samples collected in two different viral transport media were analyzed with minimal sample preparation and the subsequent mass spectra data was used to build different ML models with two different techniques. The best model showed high performance in terms of accuracy, sensitivity and specificity, in all cases reaching values higher than 90%. Our results suggest that the analysis of NP samples by MALDI-TOF MS and ML is a simple, safe, fast and economic diagnostic test for COVID-19.

## Introduction

The COVID-19 pandemic not only represents a major health crisis, but has also had unprecedented economic repercussions. According to the most recent available report of the World Health Organization (1), the cumulative number of reported cases of SARS-CoV-2 infections worldwide has now reached over 98 million people and over 2 million people have died of the disease since the start of the COVID-19 pandemic in December 2019. Moreover, it has been estimated that the gross domestic product may drop by more than 10-15% in some countries (2). Taking into consideration how rapidly the COVID-19 pandemic has spread, often through the transmission of SARS-CoV-2 by asymptomatic individuals (3), fast and economic diagnostic tools are essential for the control of this devastating pandemic.

While several diagnostic and surveillance technologies for SARS-CoV-2 have been either developed or used during the COVID-19 pandemic (4–6), real-time reverse transcription polymerase chain reaction (RT-PCR) is currently still the validated assay for early diagnosis in patients with suspected SARS-CoV-2 infection (7). However, there are certain concerns regarding RT-PCR (8) as the gold standard analytical methodology for pandemic control. PCR-based strategies are costly, require a lot of technical personnel and laboratories, and the analysis time is relatively long, limiting the number of samples that can be processed daily. Although some of these problems can be overcome by using saliva for COVID-19 diagnosis and a dual RT-qPCR test, the time needed to obtain results remains high (9, 10). Moreover, most countries do not have sufficient laboratory resources and, due to the enormous global demand, are not able to obtain a sufficient supply of PCR kits. Given this situation, new alternative methodologies need to be developed.

A useful bioanalytical methodology that would allow most of these limitations to be overcome is matrix-assisted laser desorption/ionization mass spectrometry coupled to a TOF analyzer (MALDI-TOF MS). This technique is the current tool for rapid, accurate, and cost-effective identification of cultured bacteria and fungi in clinical microbiology (11, 12) and even though it is not routinely used in hospitals to identify viruses, it has been shown to be useful for this purpose (13, 14). Although some MALDI-TOF MS methodologies need time-consuming sample preparation, such as protein or nucleotide extraction, simpler protocols are possible. For example, protocols that consist only of the mixing of a diluted sample with the matrix have been successfully applied to differentiate samples from myeloma patients and healthy subjects (15). In that study, the analyses of biological samples by MALDI-TOF MS allowed the patterns or fingerprints of different biological samples to be obtained, which could be further used to differentiate between samples (e.g., control vs. disease samples).

The large amount of data obtained using MS spectra fingerprints requires the combination of powerful statistical strategies (13) and artificial intelligence methods in order to be able to identify the diagnostic pattern. These strategies have been successfully applied in medicine and biomedicine (15, 16). The fingerprint (pattern recognition) approach avoids tedious biological sample work and eliminates the need to identify biomarkers, so considerably reducing the analysis time (17). With regards to the use of biomarkers, it should also be noted that single biomarkers are generally considered as insufficient and so it is often necessary to search for a combination of several different biomarkers to perform effective clinical diagnosis (18, 19).

A method based on recording the MALDI mass spectra of nasal swab samples previously tested for SARS-CoV-2 by RT–qPCR and their subsequent analysis by machine learning (ML) has recently been proposed for large-scale SARS-CoV-2 testing (20). In this study, samples were analyzed after adding a CHCA solution as a matrix and irradiating the MALDI plates with an ultraviolet light for 20 min to inactivate the viruses. However, in addition to the problems regarding this methodology discussed by SoRelle et al. (21), other aspects such as the application of safer sample inactivation protocols, the use of different viral transport media and the development of more robust machine learning protocols have required further progress.

In light of the above, the present work has aimed to develop a new methodology based on MALDI-TOF MS analyses of nasopharyngeal samples coupled to methods of artificial intelligence, allowing COVID-19 to be identified. We have employed a variety of machine learning approaches to analyze the pattern spectra (fingerprint) of nasopharyngeal samples in the two most widely used types of virus transport media. The methodology developed as a result of these approaches is a promising tool not only in the battle to control the spread of COVID-19 but also for post-pandemic testing in local settings to prevent future major outbreaks.

The hypothesis of the present work was that there is a MALDI-TOF mass spectral pattern (fingerprint) that can be assigned as a signature accurately characterizing negative and positive samples for SARS-CoV-2 infection. The application of a machine learning (ML) approach to the fingerprint mass spectra of positive and negative samples of SARS-CoV-2 infection will allow the development of a fast and efficient approach to support clinical decisions.

## Materials and Methods

### Chemicals

Sinapinic acid was used as a matrix for MALDI-TOF MS analysis and was purchased from Bruker Daltonics (Bremen, Germany; #8201345). Trifluoroacetic acid (TFA) was purchased from Scharlab (#AC31420100; peptide synthesis grade) and acetonitrile (mass spectrometry grade) was purchased from VWR (#83640.29). Protein Calibration Standard I (#8206355) was used for MALDI-TOF MS calibration and was purchased from Bruker Daltonics (Bremen, Germany).

### Sample Collection

A total of 237 nasopharyngeal samples were provided by the ICS-IAS Girona Clinical Laboratory (Parc Sanitari Martí i Julià; Salt, Catalonia, Spain), according to IDIBGI Biobank (Biobanc IDIBGI, B.0000872) agreement, to carry out the present study, which was approved by the Clinical Research Ethics Committee of the Doctor Josep Trueta Hospital in Girona (ref#2020.088). A consecutive non-probabilistic model was chased since samples were provided when available at several time points during the first COVID19 wave (April–July of 2020). Such samples were provided either in DeltaSwab ViCUM (#304273; DeltaLab) or DeltaSwab Virus (#304295; DeltaLab) virus transport medium. Concretely 149 samples were provided in DeltaSwab ViCUM and 88 in DeltaSwab Virus. Before delivering, they were processed in the Molecular area of the territorial laboratory of Girona under biosafety II conditions to perform both the chemical inactivation and the PCR diagnostics. The sample inactivation was performed using Ribospin vRD Buffer VL (GeneAll, Korea), which its principal component is guanidine thiocyanate in a concentration of 60–70%, by mixing 300 ul of the buffer with 300 ul of the transport medium where the NP sample was collected. The RT-PCR diagnosis was performed using two different methodological platforms which detect 2 targets (N and E qRT-PCR methodology, Xpert SARS-CoV-2, Cepheid, US) or 4 targets (N, E, S and RpRd genes, Allplex 2019-nCoV assay, Seegen, South Korea). After sample inactivation and the RT-PCR analysis samples were send to the laboratory of NEOMA research group.

### Sample Preparation for MS

Inactivated samples, previously tested by RT-PCR in the ICS-IAS Girona Clinical Laboratory, were then processed for MS analysis in the laboratory of NEOMA research group of the University of Girona.

All samples were first diluted 10 times with Milli-Q water. Theyhe were then mixed in 1:1 ratio with a solution of sinapinic acid (SA) containing 20 mg SA/mL in 60%:40% (v/v) acetonitrile (ACN): milli-Q water with 0.3% trifluoroacetic acid (TFA). TFA was added in order to increase the ionization. Finally, 1 ul of the mixture was spotted on a purified stainless-steel target plate (MTP 384 target ground steel; Bruker Daltonics, Bremen, Germany) in triplicates and allowed to dry at room temperature before being analyzed by MALDI-TOF MS. To avoid carry-over contamination, the target plate was regularly cleaned in an ultrasonic bath using a specific cleaning procedure with ultrapure solvents sequentially in this order: 2-propanol, MilliQ water, 2-propanol and TA30 (350 ml ACN: 350 ml TFA 0.1%).

### Acquisition of Mass Spectra

Mass spectra were acquired using Autoflex maX with Time-Of-Flight (TOF) analyzer from Bruker Daltonics (Bruker Daltonics, Bremen, Germany). Ionization was achieved by irradiation with a solid phase laser (with patented Smartbeam technology) operating at 2,000 Hz. All spectra were acquired automatically using a regular raster (in random walk mode) and 20 shots were made in each raster spot; locations were calibrated prior to each run. Sample mass spectra was the sum of 1,800 satisfactory shots taken in 300 shots steps. All measurements were carried out in a positive linear mode and each spectrum was externally calibrated using a standard mixture of peptides (Standard Protein I, Bruker Daltonics). All mass spectra were acquired using FlexControl software (Bruker Daltonics, Germany) and each spectrum consisted of more than 25,000 m/z values with the corresponding intensities in the mass range from 5 to 20 kDa. The smoothing of mass spectra by Savitzky-Golay method, the baseline subtraction by Top-Hat method and the recalibration of each mass spectrum was performed using the FlexAnalysis 3.4 software (Bruker Daltonics, Germany). The same software was used to export all the m/z values with the corresponding intensities into ASCII format for its further analysis using machine learning approaches.

### Machine Learning

To classify the positive and negative samples a machine learning approach was adopted. The learned model represents the best solution given the data samples obtained by MALDI-TOF MS. To study the performance, two of the most well-known and successfully applied techniques were selected. Extreme Gradient Boosting Trees (XBOOST) (22) and Support Vector Machines (SVMs) (23) were tested using different parameters to obtain their best results to the problem. A cross-validation (CV) was applied to study the performance of both XBOOST and SVM. In standard CV, instances are distributed randomly into CV partitions. But our study involved three replicas of the same sample and they were related. Therefore, in this study we considered the 3 replicas of the same individual as a unique sample in the CV phase. Also, a number of K = 10-folders was used because the number of samples was small, but a minimum of four positive and negative examples were added as a constraint for each fold to ensure that there are samples of at least two different individuals. Note that previous to the CV process, 10% of the samples were randomly separated to perform the test using the best model selected using CV. The test was done 20 times to avoid bias in the results and guarantee that the results are independent of the samples selected in the training phase (double-blind test).

Because the number of available features was large (29,393 m/z values) it is expected that many of them were highly correlated and some of them may contain irrelevant information. To overcome some of the problems that arise using high-dimensional data, all the experiments were performed using principal component analysis (PCA) as dimensionality reduction technique. Then, to analyze what was the optimal number of dimensions, the SVM and XBOOST methods were evaluated for 5, 10, and 50 projected features.

Both SVM and XBOOST have some hyper-parameters that require tuning in order to improve results. Three hyperparameters were fitted:

- Number of estimators (XBOOST): 50 and 10
- Tree depth (XBOOST): 5 and 10
- Subsample (XBOOST): between 50 and 80% in steps of 10%
- Learning rate (XBOOST): 0.01 and 0.05
- Kernel (SVM): radial base and linear functions
- Gamma (SVM): 0.01, 0.001 and 0.0001
- C-penalty parameter (SVM): 1 and 10

In order to tune the hyper-parameters, a systematic procedure known as grid-search was used. This method tries all possible combinations of hyper-parameter values. Models for each hyper-parameter combination are trained with the training partition and evaluated with the validation partition. The best combination on the validation set is selected.

Finally, F1-Score was used as a performance measure (Equation 1) to select the most suitable developed model. This criterion is very appealing when the positive and negative classes are unbalanced and we are interested in minimizing the probability that a random positive sample is included into the negative sample classification area and vice versa.

(1) $$\begin{array}{l} \text{F}1-\text{score}=2\;*\text{Precision}*\text{ Recall}/\left(\text{Precision}+\text{Recall}\right)\\ \text{Precision}=\text{TP}/\left(\text{TP}+\text{FP}\right)\\ \begin{array}{l} \text{Recall}=\text{TP}/\left(\text{TP}+\text{FN}\right) \end{array} \end{array}$$

## Results and Discussion

Based on the hypothesis of differences in the fingerprints of positive and negative samples, a machine learning (ML) approach was adopted to build a model for the fast and efficient classification of these two groups of samples. Different experiments were designed to test the ability of MALDI-TOF MS to extract these spectral patterns from the data obtained from human nasopharyngeal (NP) and enable it to detect patients infected by SARS-CoV-2.

### The Optimization of the MALDI Parameters

It is well-known that several factors can influence the results obtained by MALDI-TOF MS, including matrix and sample preparation (24). Moreover, finding the mass range in which the most relevant information can be found is also an important step. Therefore, the optimization of the methodology was first carried out with the objective to find the optimal matrix, sample dilution and mass range in order to develop a simple procedure that would allow the maximum number of peaks to be obtained with acceptable resolutions and intensities. Firstly, undiluted samples were analyzed but no signals at different mass-to-charge (m/z) values were observed in the mass spectra. When samples were diluted 10 times, rich mass spectra with acceptable resolution and intensities were obtained. Since the idea was to develop a simple and fast methodology, the analysis of the low mass range (<1,000 Da) was discarded due to the high background noise generated by the matrix (25, 26). Finally, we selected the mass spectra in the 5 to 20 kDa range. This mass spectra range was also used by Nachtigall et al. (20) in developing a similar methodology. In contrast we decided to use Sinapinic Acid (SA) for MALDI TOF MS sample analysis as this is particularly recommended for larger mass range (27).

### First Experiment: Using All Collected Samples

Initially, the transport media in which the nasopharyngeal (NP) samples were collected was not considered as a main experimental criterion. In other words, all spectra acquired from samples collected in either DeltaSwab-ViCUM or DeltaSwab-Virus transport media were used without splitting samples by the transporter. Therefore, different strategies of ML analysis were performed using the whole dataset composed of the m/z values, in the 5 to 20 kDa range, and their corresponding intensities for each sample. A total of 708 mass spectra were obtained by MALDI-TOF analysis, corresponding to samples that were previously analyzed by RT-PCR, resulting in 180 positive and 528 negative samples for SARS-CoV-2 infection.

**Figure 1:**
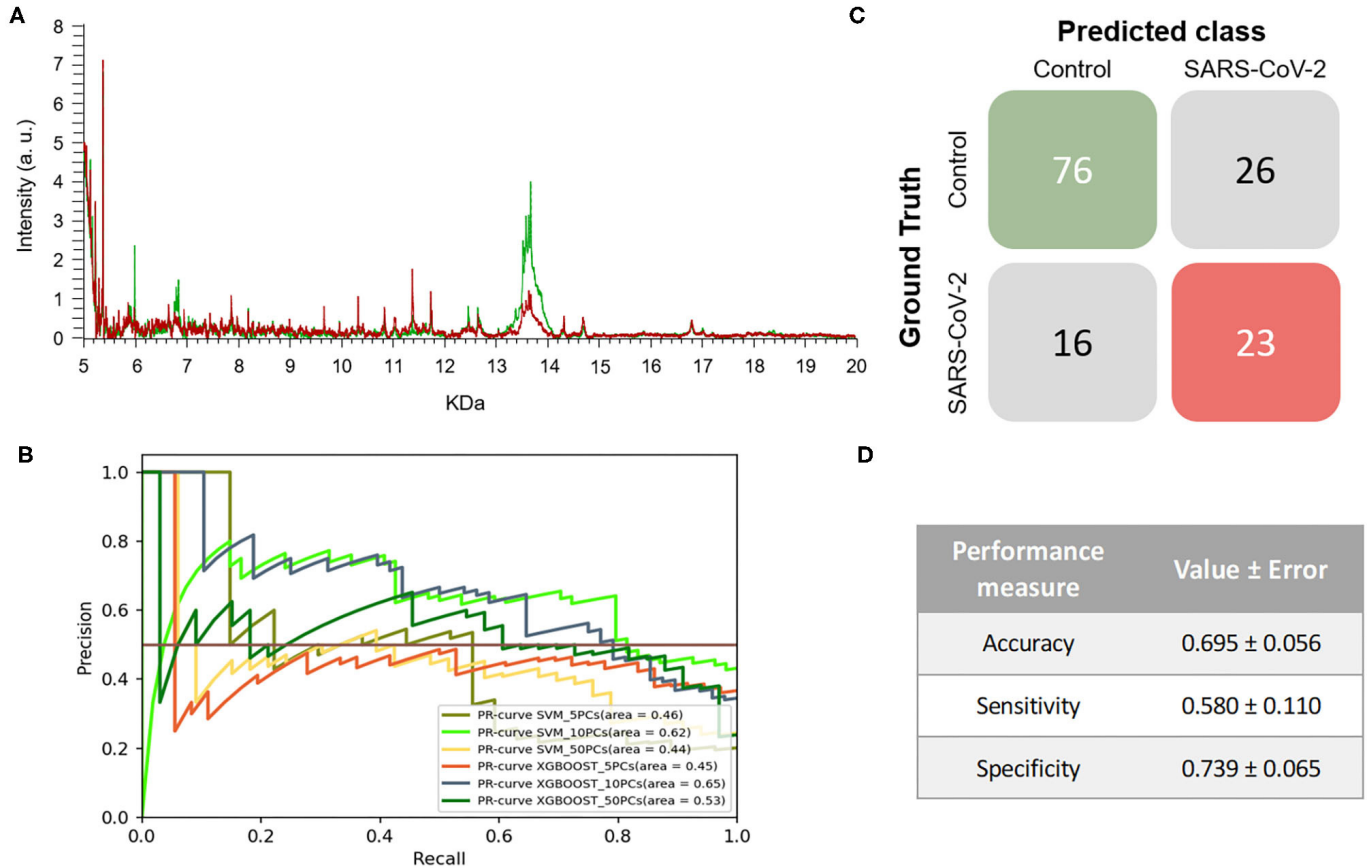
Results of the first experiment. **(A)** Representative mass spectra of NP samples for SARS-CoV-2: positive (red) and negative (green). **(B)** Precision-recall curve from the different models. **(C)** Average confusion matrix and **(D)** average performance metrics including the standard deviation (note that the test was performed 20 times selecting random different samples for each iteration) of the best model (SVM + 10PCs cross-validation *K* = 10).

The mass spectra assigned by PCR as positive samples presented differences both in the intensity of signals and the m/z values in comparison to the spectra of PCR negative samples. Despite the differences exhibited, no specific biomarkers for any of the positive or negative PCR sample groups were found (Figure 1A). The ML approach was then applied to analyze the data from the entire range of the selected m/z pattern (fingerprint) without applying any variable selection method. Six models were constructed using both XGBOOST and support vector machine (SVM) algorithms with different numbers of principal components (PCs) (5, 10 or 50 PCs) to identify the conditions that would tend toward lower variance. When training the different models, performance analyses showed that the models' accuracies, sensitivities, and specificities did not vary significantly between the different number of PCs. Based on the metric F1-score, which is the harmonic-mean of precision and recall, the best model for this experiment was obtained by SVM + 10PCs. The model was able to perform better than baseline (F1-Score = 0.639 ± 0.056) (Table 1), showing the existence of a general pattern associated to the mass spectra. These results can also be observed in the precision-recall (PR) curve (Figure 1B), providing insights that suggest the best model. Overall, the resulting model reached an 0.580 ± 0.110 and 0.739 ± 0.065 of sensitivity and specificity, respectively. This model was then applied to perform the test process. Figures 1C,D shows the matrix confusion of this experiment with the summary of the predicted results for all the samples used to perform the test.

**Table 1:** Model learning (cross validation results *K* = 10) of all the models tested in the different experiments.

	**PCs used**	**F1-Score**		**Sensitivity (TPR)**		**Specificity (TNR)**		**Accuracy**	
		**XGBOOST**	**SVM**	**XGBOOST**	**SVM**	**XGBOOST**	**SVM**	**XGBOOST**	**SVM**
Experiment 1	*n* = 5	0.625 ± 0.041	0.595 ± 0.073	0.658 ± 0.093	0.780 ± 0.115	0.684 ± 0.062	0.580 ± 0.113	0.677 ± 0.043	0.626 ± 0.078
	*n* = 10	0.620 ± 0.043	**0.639** ± **0.056**	0.580 ± 0.084	0.580 ± 0.110	0.716 ± 0.066	0.739 ± 0.065	0.678 ± 0.043	0.695 ± 0.056
	*n* = 50	0.587 ± 0.045	0.558 ± 0.057	0.283 ± 0.098	0.238 ± 0.094	0.890 ± 0.055	0.891 ± 0.033	0.679 ± 0.065	0.703 ± 0.048
Experiment 2 (VTM1 results)	*n* = 5	0.749 ± 0.066	0.759 ± 0.086	0.538 ± 0.168	0.525 ± 0.150	0.946 ± 0.033	0.950 ± 0.051	0.852 ± 0.039	0.832 ± 0.070
	*n* = 10	0.793 ± 0.116	**0.826** ± **0.088**	0.646 ± 0.242	0.652 ± 0.176	0.939 ± 0.070	0.969 ± 0.031	0.884 ± 0.069	0.879 ± 0.065
	*n* = 50	0.754 ± 0.132	0.700 ± 0.160	0.548 ± 0.275	0.487 ± 0.319	0.961 ± 0.058	0.937 ± 0.071	0.876 ± 0.075	0.845 ± 0.080
Experiment 2 (VTM2 results)	*n* = 5	0.433 ± 0.080	0.418 ± 0.104	0.273 ± 0.081	0.285 ± 0.200	0.618 ± 0.119	0.588 ± 0.196	0.494 ± 0.082	0.491 ± 0.139
	*n* = 10	0.482 ± 0.106	**0.515** ± **0.110**	0.241 ± 0.162	0.381 ± 0.206	0.762 ± 0.138	0.688 ± 0.133	0.581 ± 0.111	0.594 ± 0.107
	*n* = 50	0.511 ± 0.120	0.476 ± 0.093	0.158 ± 0.172	0.135 ± 0.148	0.914 ± 0.063	0.925 ± 0.075	0.711 ± 0.083	0.654 ± 0.098
Experiment 3	*n* = 5	0.891 ± 0.109	**0.979** ± **0.048**	0.922 ± 0.127	0.974 ± 0.076	0.872 ± 0.157	0.988 ± 0.054	0.897 ± 0.109	0.980 ± 0.044
	*n* = 10	0.950 ± 0.050	0.947 ± 0.066	0.930 ± 0.070	0.987 ± 0.040	0.970 ± 0.090	0.918 ± 0.110	0.981 ± 0.052	0.950 ± 0.063
	*n* = 50	0.868 ± 0.109	0.968 ± 0.055	0.911 ± 0.106	0.989 ± 0.484	0.832 ± 0.214	0.941 ± 0.118	0.882 ± 0.093	0.972 ± 0.045
Robustness analysis	*n* = 5	0.647	**0.964**	0.956	1.000	0.377	0.923	0.687	0.964
	*n* = 10	0.609	0.945	0.922	0.964	0.346	0.923	0.654	0.945
	*n* = 50	0.690	0.902	0.956	1.000	0.446	0.795	0.719	0.902

*Bold values represent the best model for each experiment using the selected metric F1-score*.

The results demonstrated that the developed a methodology, without considering the viral transport media, enables SARS-CoV-2-positive and -negative samples to be discriminated with around 70% accuracy, 60% sensitivity and 74% specificity. Although these percentages may be lower than the expectations, it is worth noting that RT-PCR for COVID-19 diagnosis only reaches clinical sensitivities of between 38 and 78% (28, 29). In the case of RT-PCR in nasopharyngeal swabs, sensitivity has not been found to exceed 70% (30, 31). Therefore, our first developed model using two different transport media would be as useful as RT-PCR in monitoring the spread of COVID-19 during a pandemic in which both incidence and prevalence are high. It is important to note that the mass spectra of the samples used to train and develop the different ML models were classified in the control group and in the COVID-19 group based on the previous RT-PCR result. However, there is a high rate of false negatives in RT-PCR results, estimated at between 2 and 29% by Arevalo-Rodriguez et al. (32), which may hinder the learning, validation and testing steps of the classification model. The drawbacks of RT-PCR as a diagnostic test for COVID-19 and the lack of a clear gold-standard complicates the evaluation of new methodologies (8, 33, 34).

Interestingly, the optimized model for the prediction of SARS-CoV-2-positive and negative samples shows a greater ability to detect negative samples than positive ones (despite the standard deviation of the sensitivity being quite large for the different tests performed). These results may indicate that the developed model has a dependence on factors such as the heterogeneity of the samples and the noise of the data. This heterogeneity of the data in this first experiment may be caused by the different viral transport media used for sample collection. The high demand during the COVID-19 pandemic for the specific transport media for SARS-CoV-2, resulted in the use of alternative viral transport media, following the recommendations of the FDA, that had the effect of increasing the heterogeneity of the samples received in the laboratories (35).

### Second Experiment: Splitting Samples by the Viral Transport Media

To test the hypothesis that the heterogeneity of the data was a result of the different viral transport media that were used, the dataset was split by DeltaSwab-ViCUM (viral transport medium 1, VTM1) and DeltaSwab-Virus (viral transport medium 2, VTM2).

**Figure 2:**
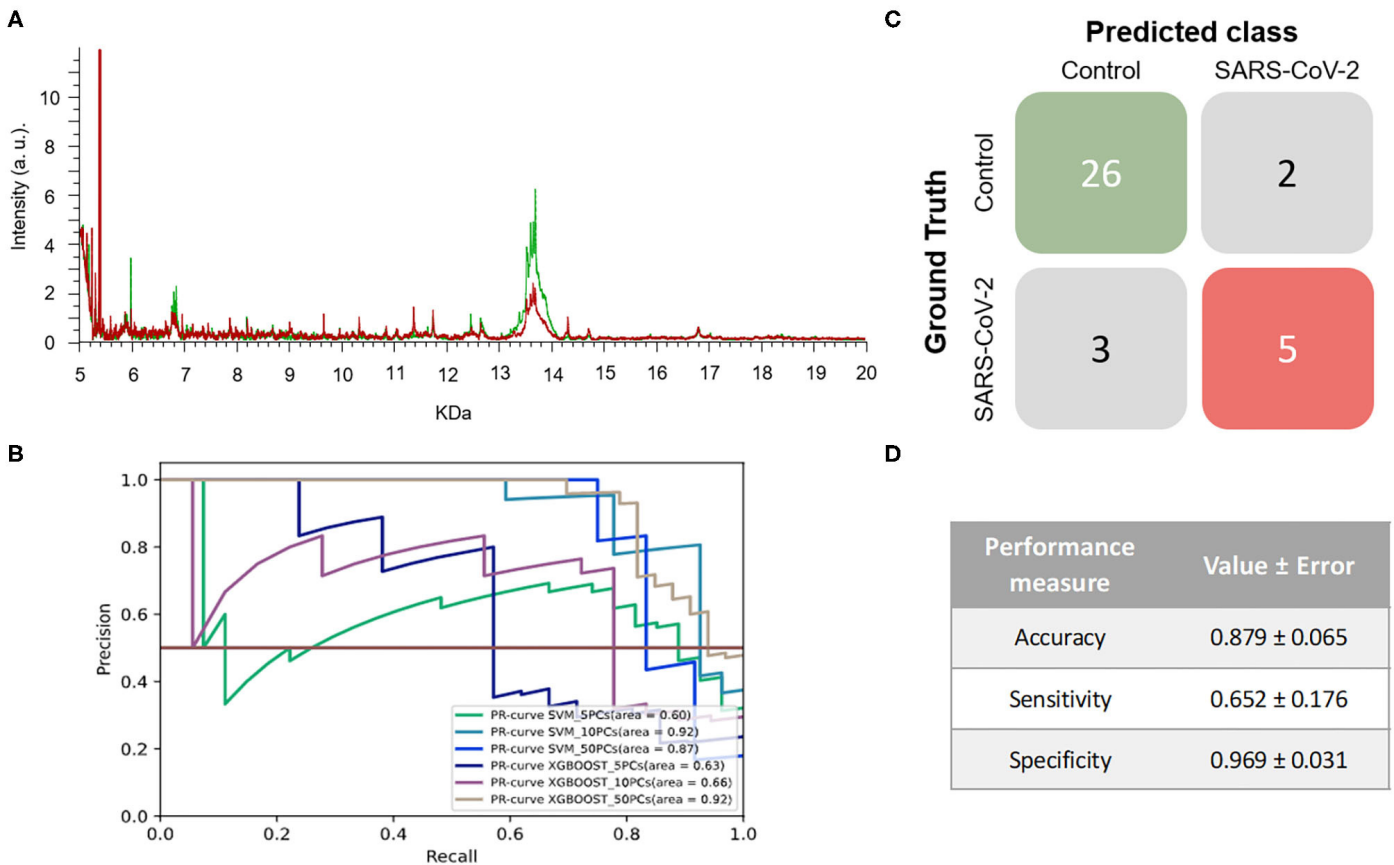
Results for DeltaSwab-ViCUM (VTM1). **(A)** Representative mass spectra of NP samples for SARS-CoV-2: positive (red) and negative (green). **(B)** Precision-recall curve from the different models. **(C)** Average confusion matrix and **(D)** average performance metrics including their standard deviation (the test was performed 20 times selecting randomly different samples for each iteration) of the best model (SVM + 10PCs cross-validation *K* = 10).

As for VTM1, 443 mass spectra (111 and 332 from samples that were positive and negative for SARS-CoV-2, respectively) were obtained from NP samples collected in this viral transport media. As observed in the first experiment, the spectra of the two groups present differences in certain regions (Figure 2A). The same ML strategy as was used in the previous section was then applied to analyze the information from the corresponding pattern of the mass spectra (fingerprint). The results did not vary substantially between the different ML models applied to perform the analysis (Figure 2B and Table 1). However, of the different models tested the best results in terms of the F1-score were obtained when the SVM using 10 PCs (F1-score = 0.826 ± 0.088) was applied (Table 1).

**Figure 3:**
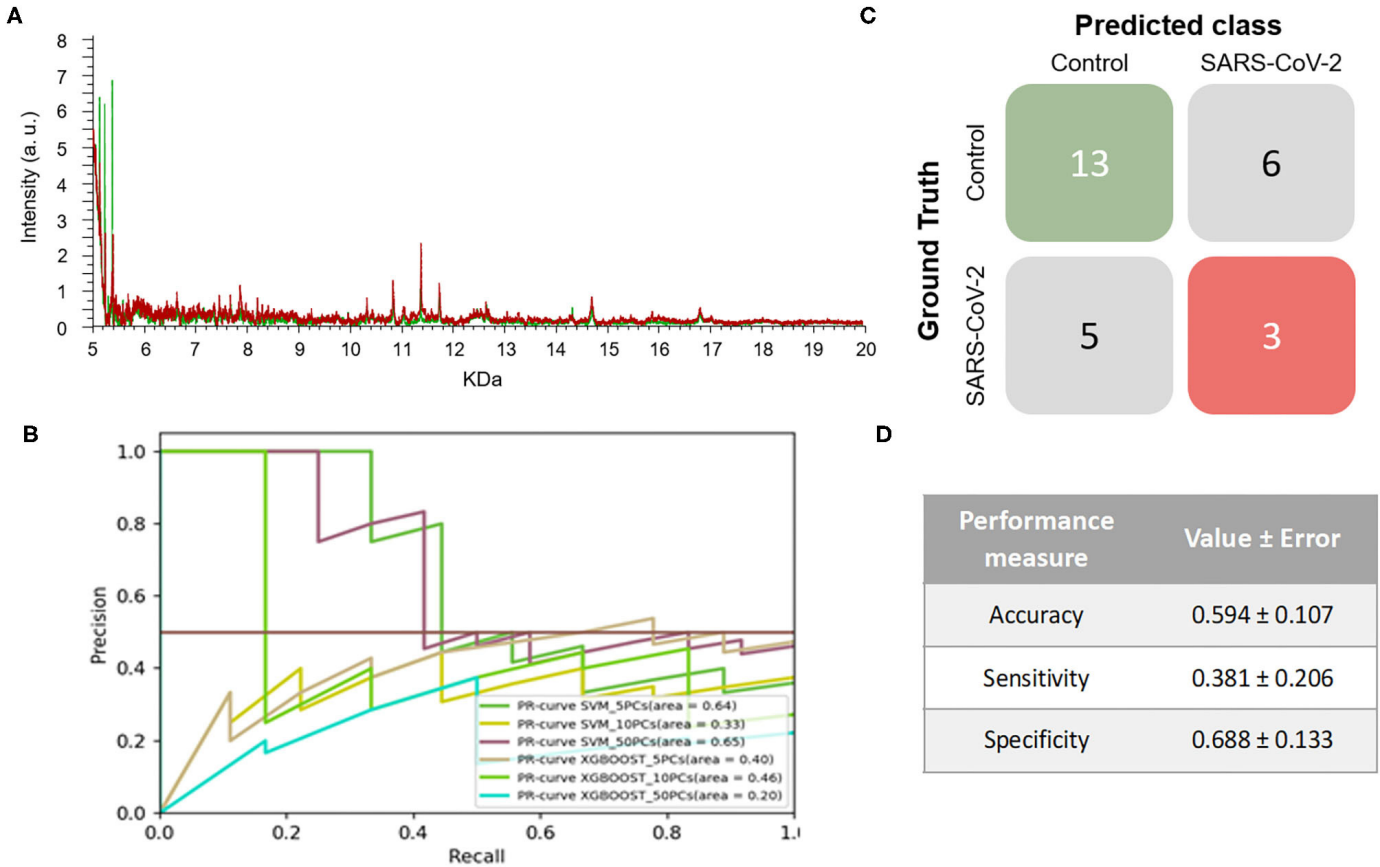
Results for DeltaSwab-Virus (VTM2). **(A)** Representative mass spectra of NP samples for SARS-CoV-2: positive (red) and negative (green). **(B)** Precision-recall curve from the different models. **(C)** Average confusion matrix and **(D)** average performance metrics including their standard deviation (the test was performed 20 times selecting randomly different samples for each iteration) of the best model (SVM + 10 PCs cross-validation *K* = 10).

On the other hand, 264 mass spectra [69 positive and 195 negative for SARS-CoV-2, respectively] were obtained from NP samples collected in VTM2. In this case, no clear differences were observed between the spectral pattern of positive and negative samples (Figure 3A). After analyzing the spectral data by the different ML models, the best model was obtained for SVM using 10PCs (F1-score = 0.515±0.110) (Figure 3B and Table 1).

The results obtained relating to the performance of the two best models clearly differed. Accuracy (VTM1 = 0.879 ± 0.065 vs. VTM2 = 0.594 ± 0.107), sensitivity (VTM1 = 0.652 ± 0.176 vs. VTM2 = 0.381 ± 0.206), and specificity (VTM1 = 0.969 ± 0.031 vs. VTM2 = 0.688 ± 0.133) all varied significantly between the two transport media (Figures 2C,D, 3C,D). The model that gave the best results was the one with VTM1, reaching 88, 65, and 97% for accuracy, sensitivity and specificity, respectively. The results prove that these two virus transporters behave differently and that VTM1 is better suited for the detection of SARS-CoV-2 infected samples. This finding that the model is reagent-dependent is unsurprising as the use of different analytical platforms in clinical laboratories require specific sample collectors and reagents in order to obtain accurate results.

Analyzing the results more deeply, it can be seen that despite the promising results related to VTM1 in this second experiment, there was still a significant standard deviation value in the sensitivity (±0.176), suggesting either internal VTM1 heterogeneity and noisy samples. This heterogeneity might be due to the nucleic acid amplification technique used for their analysis since different PCR platforms were used in the clinical laboratory. The main difference between the used platforms in this study lies in the number of the targets detected by PCR amplification related to viral structural proteins. At that time of the pandemic that we collected the samples, two targets (N and E qRT-PCR methodology, Xpert SARS-CoV-2, Cepheid, US) or four targets (N, E, S and RpRd genes, Allplex 2019-nCoV assay, Seegen, South Korea) were detected, considering both platforms methodologically equivalents. However, these platforms provide positive or negative results for the SARS-CoV-2 detection by using different diagnostic algorithms (true positive is considered when we observe at least two targets amplified). It has to be highlighted that the sensibility and the specificity of the RT-PCR is assay-dependent (6, 30, 33, 36). So, it is important to remark that the analytical sensitivity and specificity of these platforms, which are both qualitative, can change due to the difference in the number of the targets detected.

Despite this methodological platform variability, we hypothesize that the greatest source of heterogeneity might be the moment during the pandemic at which the samples were collected. It is known that disease prevalence plays an important role in the accuracy of a specific test (33, 37, 38). The prevalence of COVID-19 was different during the peak of the pandemic than at the beginning of the de-escalation phase when the incidence of infections was much lower. Remarkably, the available number of SARS-CoV-2-positive samples for analysis was extremely low when compared with the negative ones. As a result, the class imbalance observed in the dataset was due to the low number of samples collected at the beginning of the de-escalation phase.

In light of the above, we decided to split the samples collected in viral transport media 1 into two groups based on the moment during the pandemic at which they were collected. Hence, in a third experiment, samples collected during the peak of the pandemic period were used for ML calculations due to their greater homogeneity, while those collected later during the de-escalated phase when the incidence of infections was much lower were discarded for this purpose.

### Third Experiment Focusing on DeltaSwab-ViCUM Transport Media

For this third experiment, NP samples collected in VTM1 between April and the beginning of May were analyzed. A total of 173 mass spectra (89 and 84 from samples that were positive and negative for SARS-CoV-2, respectively) were obtained and analyzed in this experiment.

**Figure 4:**
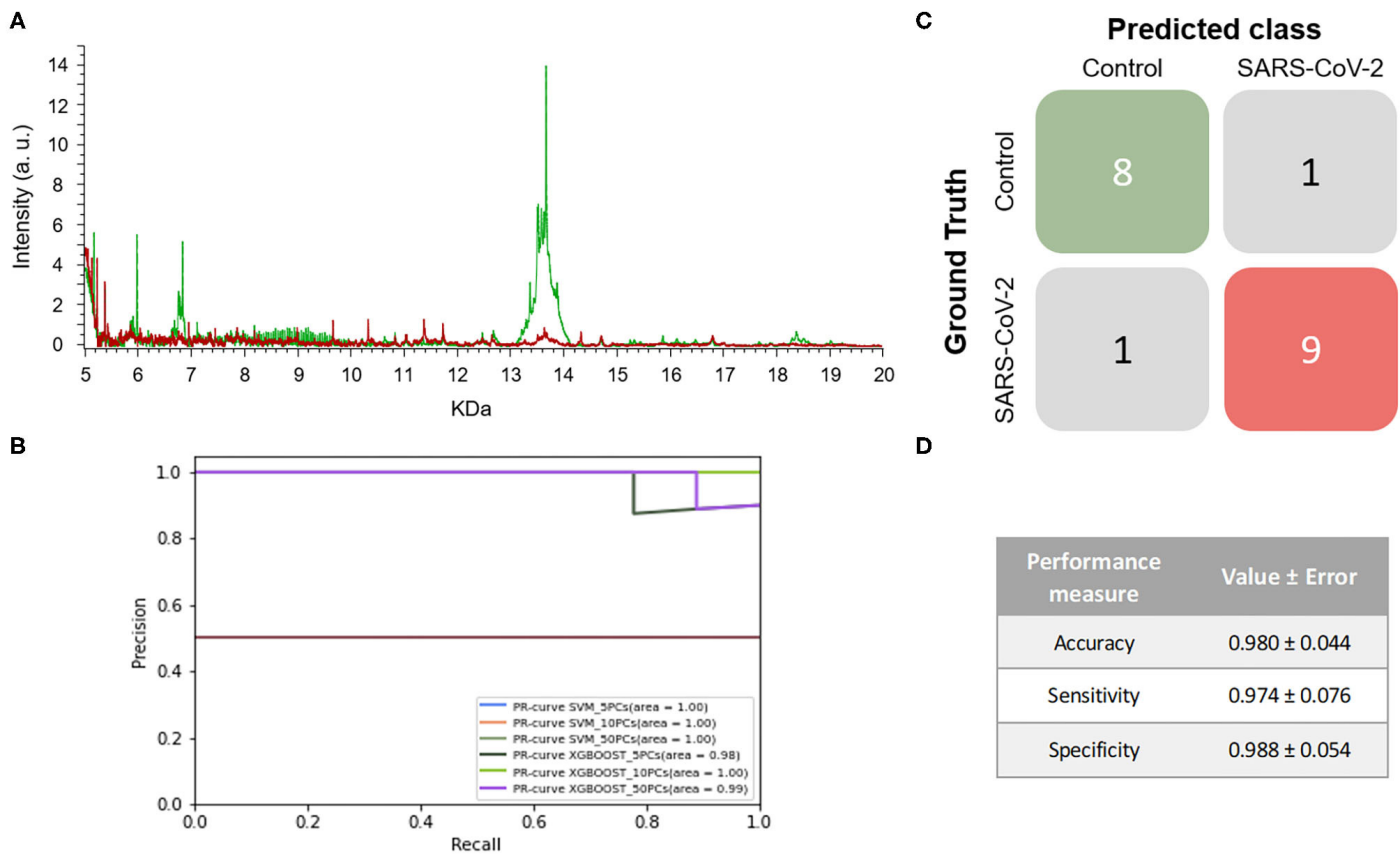
Results for DeltaSwab-ViCUM (VTM1) within the high incidence pandemic period. **(A)** Representative mass spectra of NP samples for SARS-CoV-2: positive (red) and negative (green). **(B)** Precision-recall curve from the different models. **(C)** Average confusion matrix and **(D)** average performance metrics including their standard deviation (the test was performed 20 times selecting randomly different samples for each iteration) of the best model (SVM + 5PCs cross-validation *K* = 10).

When the mass spectra of positives NP samples were compared with the mass spectra of the negative ones, noticeable differences were observed in the profile of the mass spectra (Figure 4A). None of the performance measures varied significantly with the different numbers of PCs selected nor with the different ML approaches. Although all of the models showed good results, the one with the highest F1-score was the SVM using 5 PCs (F1-score = 0.979 ± 0.048) and so this was considered to be the best model (Figure 4B and Table 1). These results were not only highly precise but also had low variability, demonstrating that the learned models are independent of the training data selected and valid for SARS-CoV-2 infection detection in future samples. The average confusion matrix (Figures 4C,D) shows the high performance obtained for both negative and positive samples in terms of accuracy, sensitivity and specificity, in all cases reaching values higher than 90% (0.981 ± 0.052, 0.993 ± 0.040, and 0.974 ± 0.085, respectively).

Considering the promising results obtained in this third experiment, a final experiment was performed to evaluate the robustness of the methodology in which the results of an independent set of samples were tested.

### Robustness Analysis and Applicability

As has been said, two different sample sets were acquired during the peak of the pandemic. Since the final objective is to develop a methodology that is able to discriminate between a SARS-CoV-2-positive and -negative sample, we needed to test whether an independent data set was correctly classified using the model that was built using a different sample set.

With all the previous results described above, we decided to test the methodology using the same samples as in the third experiment, consisting of NP samples collected in VTM1 during the peak of the pandemic but selecting different time periods to train and test. Thereof, these group of samples consists of two independent sample sets. The first set (Set 1; S1) consisted of NP samples collected in VTM1 at the end of April 2020 while the second set (Set 2; S2) corresponded to those samples collected in the same transport media at the beginning of May. Unlike the third experiment where all samples were considered as a single group, in this experiment the 89 mass spectra (45 positive and 44 negative) of the samples of set 1 (S1) were used to train a model and the 84 mass spectra (45 positive and 39 negative) of set 2 were used to test it.

**Figure 5:**
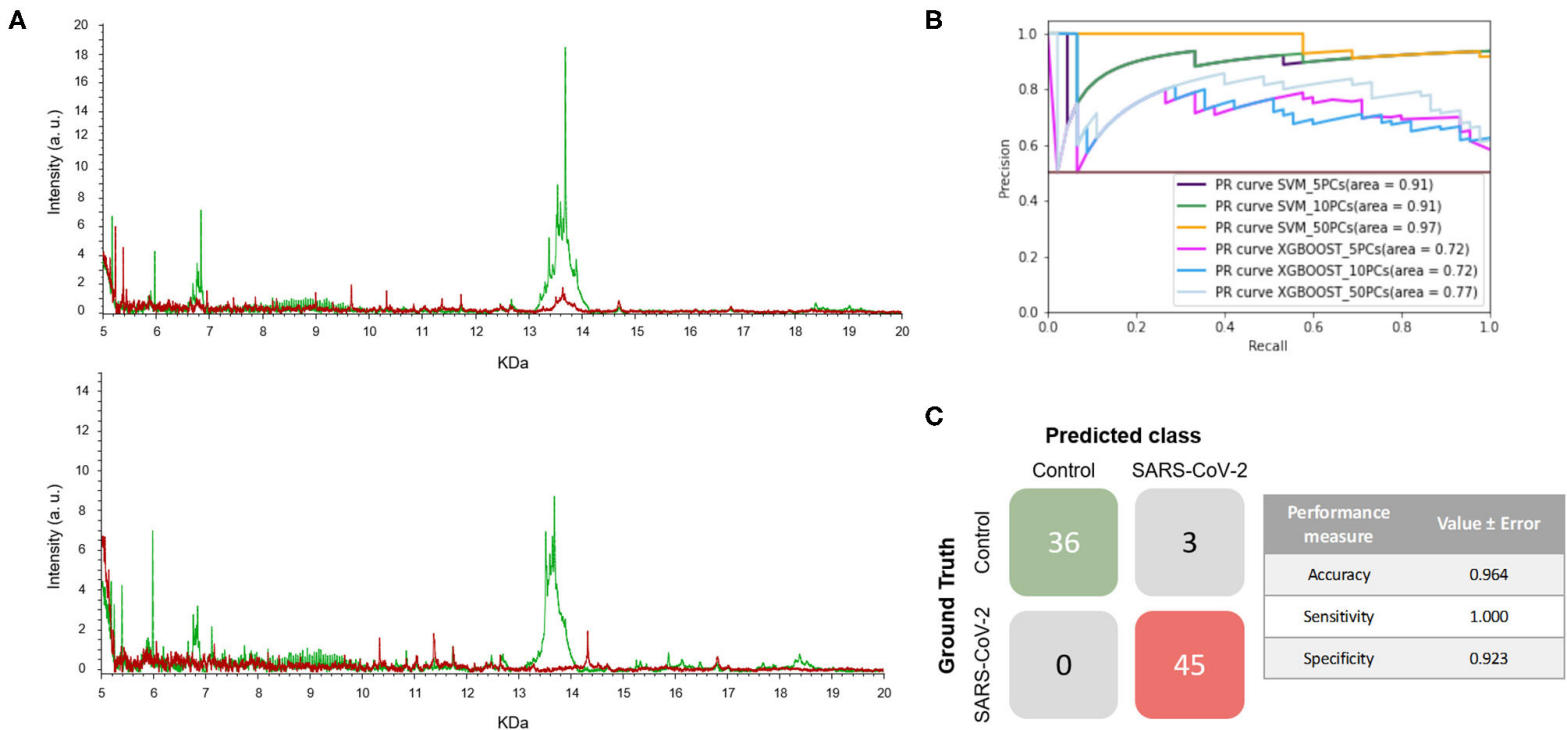
Developed methodology robustness. **(A)** Representative mass spectra of NP samples [positive (red) and negative (green)] for SARS-CoV-2 of S1 (top mass spectra) and S2 (down mass spectra). **(B)** Precision-recall curve from the different models. **(C)** Matrix confusion showing the summary of the results of all the samples used in the test phases and performance measures of the best model (SVM + 5PCs cross-validation *K* = 10). Note that no errors are included given that the results correspond to a single test set.

The mass spectra of the two sets of samples are shown in Figure 5A. As can be seen, the positive and negative samples for SARS-CoV-2 infection have clearly distinct fingerprints. In this experiment, the best model, which was the SVM using 5 PCs, had a F1-score of 0.964 (Figure 5B and Table 1). Excellent results were obtained in terms of accuracy, sensitivity and specificity, reaching values of 97, 100 and 92%, respectively (Figure 5C). Moreover, these high values with low variation errors showed the robustness of the developed methodology when used for the detection of SARS-CoV-2-positive samples.

Overall, our results demonstrate that the developed method represents a promising method for the detection of SARS-CoV-2-positive samples. As it can be seen, the best results are achieved using the VTM1. However, a similar methodology was also described by Nachtigall et al. (20) where another viral transport media (Cary-Blair transport medium) was used. In contrast to what we have undertaken here, they did not study neither the effect that the use of different viral transport media might have in the results nor performed double-blind test in the ML. Another difference is that the samples used in our study were chemically inactivated whereas only ultraviolet irradiation was performed in their study. This detail is of great significance given that SARS-CoV-2 is extremely infectious and working with inactivated samples not only reduces the risk of in-lab infection during the experimental procedure but also reduces the mental stress that could be experienced by laboratory staff (39). In line with our results, an earlier study has also found that using inactivated samples does not interfere with RT-PCR results (40).

Due to the high infectivity rate of SARS-CoV-2, an accurate and rapid diagnosis of both symptomatic and asymptomatic patients is needed to reduce the spread of the virus (31, 40). Different diagnostic methodologies have been developed, each with its own specific applications as well as its own its advantages and drawbacks (5). However, all these methodologies share certain limitations: low sensitivity, high rate of false negatives, high dependence on the moment of the diagnostic window in which the sample is collected, etc. (8, 28, 30, 34, 41). Given that asymptomatic infected people can also spread the virus, a rapid and economic screening test is needed to detect those SARS-CoV-2-positive patients without symptoms (3, 5, 42). Considering the cost of materials per specimen, we estimate that the cost of MALDI-TOF MS will be no more than 25% of the cost of RT-PCR analysis. With regards to the length of time required to receive results, the turn-around-time of a conventional RT-PCR in which an RNA extraction phase is also needed is around 6 h (40) whereas the analysis of the NP samples by MALDI-TOF MS takes less than a third part of this. Therefore, the use of MALDI-TOF -MS analysis, which is widely available in clinical laboratories, as a screening technique for SARS-CoV-2 infection detection will offer enormous savings both in time and cost.

As said, community prevalence and pre-test probability have an important effect on the positive and negative predictive value of a diagnostic test (37, 38). Under a clinical point of view, this pandemic situation has been one of the most challenging experiences in the laboratories; recruiting resources, reagents, methodological platforms and personal staff has been the most difficult goal to achieve for the very last months. Nowadays, this new situation is extremely demanding due to the high incidence and prevalence in the general population. Vaccines are ready to be used as immunological protection against SARS-CoV-2 (43) so we will probably notice shortly a descent of the cases. In this future and new scenario and with lower prevalence in the population, we will need a strong and accurate methodology that provides results requiring less time and investment. MALDI-TOF is the best option available in clinical laboratories that can reach this purpose. Moreover, this equipment is commonly found in clinical laboratories also in the developing countries, which means that the implementation of the developed methodology would not require a huge economic cost.

**Figure 6:**
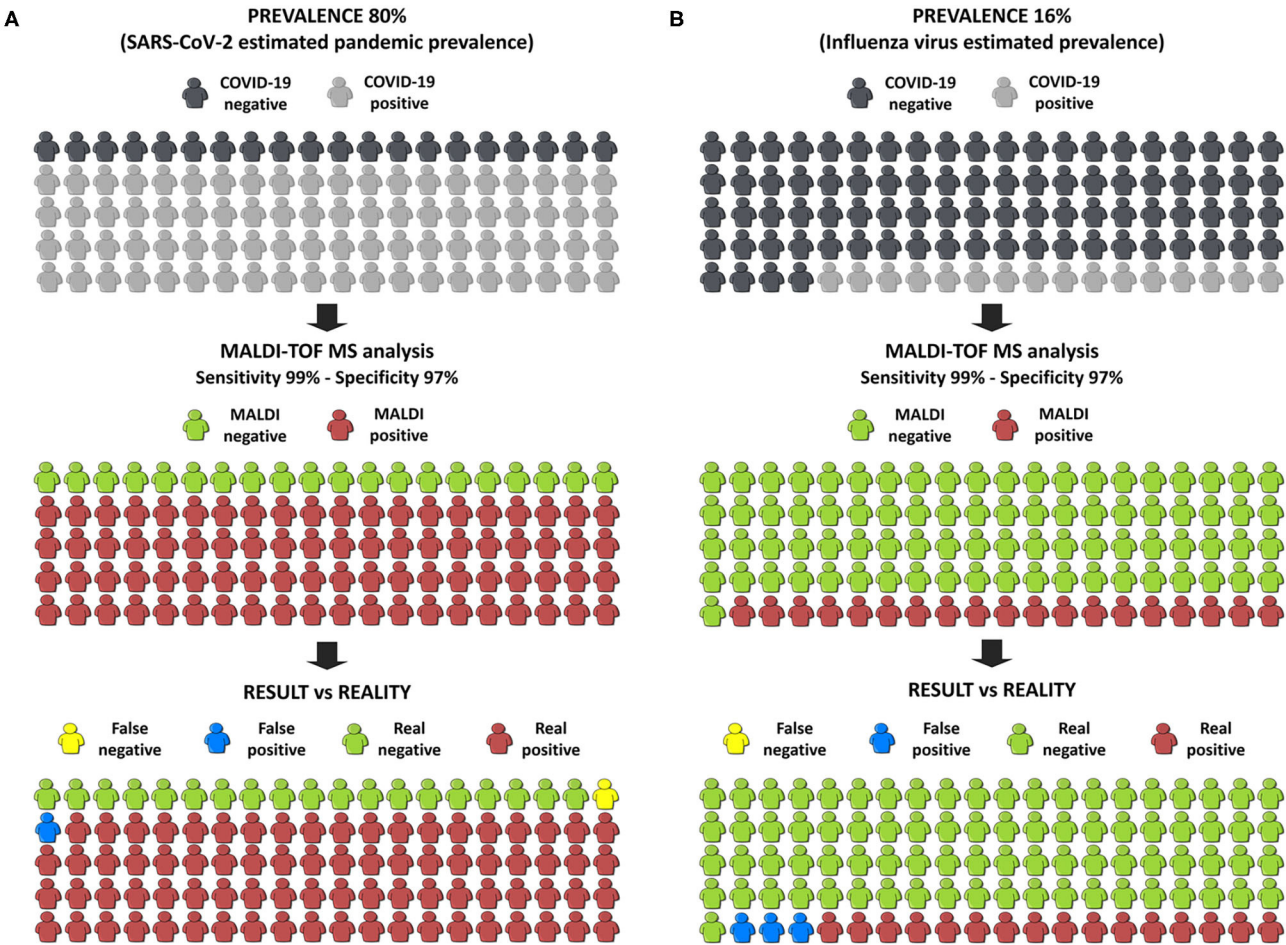
Infographic of the simulation of the results in the society. **(A)** Simulation in a pandemic situation. **(B)** Simulation in a post-pandemic situation.

Finally, in order to estimate the benefit to society of this innovation, we have used the online calculator of the BMJ (33) applying our best developed model. Firstly, a pandemic situation was simulated taking an estimated prevalence of COVID-19 in Europe of 80%, which was a pre-test probability calculated by the WHO. If 100 people were tested, and only 1 false negative and 1 false positive were obtained (Figure 6A), the probability of having COVID-19 if the test is negative is only 5%. To simulate the results in a post-pandemic situation, the pre-test probability was set at 16% which is the estimated prevalence of the influenza virus in Europe. In this case, only 3 false positives and no false negatives were obtained (Figure 6B). These two simulations strongly support our own conclusion that the developed methodology, consisting in the analysis NP samples by MALDI-TOF-MS in combination with machine learning approaches, is suitable to diagnose COVID-19 patients not only in pandemic situations but also in an epidemic situation as a screening tool in the first steps of diagnosis.

## Data Availability Statement

The datasets presented in this article are not readily available because the data that support the findings of this study are available from the corresponding author upon reasonable request. Requests to access the datasets should be directed to Pere Boadas-Vaello, pere.boadas@udg.edu.

## Ethics Statement

The studies involving human participants were reviewed and approved by Clinical Research Ethics Committee of the Doctor Josep Trueta Hospital in Girona (ref#2020.088). Written informed consent for participation was not required for this study in accordance with the national legislation and the institutional requirements.

## Author Contributions

PB-V, MD, and VS conceived the experiments, supported by EV and MS. OJ-R and MS were in charge of the NP samples inactivation and management. MALDI-MS experiments were carried out by MD and PB-V. Spectra analyses were carried out by MD, EP-M, and JC. Machine learning analyses were performed by EG-C and MD. All authors were involved in interpretation of the data and they contributed to both critical discussion of the results and elaboration of the manuscript. All authors listed above have contributed sufficiently to be included as authors.

## Conflict of Interest

The authors declare that the research was conducted in the absence of any commercial or financial relationships that could be construed as a potential conflict of interest.
